# Structural Characterization of Toxicologically Relevant Cd^2+^-L-Cysteine Complexes

**DOI:** 10.3390/toxics11040294

**Published:** 2023-03-23

**Authors:** Astha Gautam, Amanda Gomez, Emérita Mendoza Rengifo, Graham N. George, Ingrid J. Pickering, Jürgen Gailer

**Affiliations:** 1 Department of Chemistry, University of Calgary, 2500 University Drive NW, Calgary, AB T2N 1N4, Canada; 2 Molecular and Environmental Science Research Group, Department of Geological Sciences, University of Saskatchewan, Saskatoon, SK S7N 5E2, Canada

**Keywords:** cadmium, toxicological chemistry, bloodstream, complex formation, L-cysteine, chloride

## Abstract

The exposure of humans to Cd exerts adverse human health effects at low chronic exposure doses, but the underlying biomolecular mechanisms are incompletely understood. To gain insight into the toxicologically relevant chemistry of Cd^2+^ in the bloodstream, we employed an anion-exchange HPLC coupled to a flame atomic absorption spectrometer (FAAS) using a mobile phase of 100 mM NaCl with 5 mM Tris-buffer (pH 7.4) to resemble protein-free blood plasma. The injection of Cd^2+^ onto this HPLC-FAAS system was associated with the elution of a Cd peak that corresponded to [CdCl_3_]^−^/[CdCl_4_]^2−^ complexes. The addition of 0.1–10 mM L-cysteine (Cys) to the mobile phase significantly affected the retention behavior of Cd^2+^, which was rationalized by the on-column formation of mixed CdCys_x_Cl_y_ complexes. From a toxicological point of view, the results obtained with 0.1 and 0.2 mM Cys were the most relevant because they resembled plasma concentrations. The corresponding Cd-containing (~30 μM) fractions were analyzed by X-ray absorption spectroscopy and revealed an increased sulfur coordination to Cd^2+^ when the Cys concentration was increased from 0.1 to 0.2 mM. The putative formation of these toxicologically relevant Cd species in blood plasma was implicated in the Cd uptake into target organs and underscores the notion that a better understanding of the metabolism of Cd in the bloodstream is critical to causally link human exposure with organ-based toxicological effects.

## 1. Introduction

The ongoing industrialization and the concomitant large-scale emission of a variety of pollutants have become an imminent threat to public health [1]. The emission of inorganic pollutants into the environment, for example, is predominantly caused by the industrial scale mining of elements for the manufacturing of consumer goods and the consumption of unprecedented amounts of toxic metal(loid)-laden fossil fuels for their assembly. In fact, 35–77% of anthropogenic mercury emissions are attributed to the combustion of fossil fuels and wastes [2]. Owing to the inherent exceptional longevity of toxic metals in the environment [3], the chronic exposure of certain human populations is concerning [4,5], especially since the exposure to exceedingly small daily doses predominantly via the diet can severely affect human health [6,7]. To this end, research efforts are under way to address the contamination of rice with Cd to reduce the dietary exposure of humans to this highly toxic metal [8]. The urgent need to discover causal relationships between chronic human exposure to toxic metal species and diseases of unknown etiology requires new insight into the biomolecular mechanisms which govern the exposure–response relationship [9]. In this context, the metabolism of toxic metal species in the bloodstream is of fundamental importance [9], but poorly understood, in large part because of the underlying biological complexity [10].

Regarding the bloodstream, there are two conceptually distinct bioinorganic processes of toxic metal species that are of toxicological relevance. The first are processes that unfold within red blood cells [11] and the second are those processes which occur in plasma and are of indirect toxicological relevance as they ‘merely’ translocate the toxic cargo to target organ cells [12]. To this end, the small molecular weight (SMW) metabolite homocysteine, which is present in blood plasma at μM concentrations [10] has recently been shown to be likely implicated in the delivery of the neurotoxin CH_3_Hg^+^ to the blood–brain barrier (BBB) [13]. While several plasma proteins are known to bind Cd^2+^ [14], the role that SMW ions and biomolecules present in blood plasma may play in the delivery of toxic metal species to target organs is less well-understood [15,16].

To better understand the toxicological chemistry of Cd in the bloodstream, we evaluated and structurally characterized the formation of Cd complexes with L-cysteine (Cys) at physiologically relevant concentrations. Cys is present in blood plasma at concentrations up to 300 µM and has long been known to have an affinity for Cd^2+^ [17]. Indeed, the formation of Cd complexes with Cys, including [CdCys]^+^, [Cd(Cys)_2_], [Cd(Cys)_3_]^−^, [Cd_2_(Cys)_3_]^+^ [18], as well as [Cd(Cys)_4_]^2−^ [19], some of which may also form at physiological pH, has been reported. In addition, Cd^2+^ is also known to form mixed Cd complexes where the metal center is coordinated to organic functional groups as well as chloride atoms [20,21]. From a purely inorganic chemistry point of view, Cd^2+^ has long been known to form [CdCl_3_]^−^ and [CdCl_4_]^2−^ complexes in aqueous solutions [22]. Given that blood plasma contains ~105 mM of Cl^−^, these species are likely to be formed albeit transiently in blood plasma. Thus, the absorption of Cd into the bloodstream is likely to result in the formation of mixed complexes in which the metal center is coordinated to Cl^−^, Cys, and/or plasma proteins. To probe the formation of toxicologically relevant Cd species at near physiological conditions, we employed a hyphenated instrumental analytical approach, namely, HPLC coupled to a flame atomic absorption spectrometer (FAAS). This approach represents a particularly useful tool for this purpose as it allows one to employ a mobile phase that resembles the physiological conditions of blood plasma and to observe changes in the retention behavior of a toxic metal as a function of increasing concentrations of a biomolecule dissolved in the mobile phase. While we have previously used size exclusion chromatography-FAAS [16], other separation mechanisms may also be employed. Considering that Cd^2+^ forms [CdCl_3_]^−^ and [CdCl_4_]^2−^ complexes at the physiological conditions of protein-free plasma (i.e., 100 mM chloride and pH 7.4) [23], anion-exchange chromatography was chosen as a feasible approach to observe the on-column formation of Cd complexes with Cys and Cl^−^. Therefore, Cd^2+^ was injected onto an AEX/HPLC-FAAS system first using a 100 mM chloride and 5 mM Tris-buffer pH 7.4 mobile phase, and then the Cys concentration was gradually increased. The observed changes of the retention behavior of Cd^2+^ were intended to obtain new insight into the formation of Cd^2+^/Cys/Cl complexes at near physiological conditions, which may contribute to establishing the entire sequence of bioinorganic processes that mediate the Cd^2+^ uptake into its toxicological target organs.

## 2. Experimental Methods

### 2.1. Chemicals and Solutions

Tris [2-Amino-2-hydroxymethyl)-1,3-propanediol; 99.5], NaCl (>99.5%), L-cysteine (Cys, ≥98% purity), and CdCl_2_ (99.99%) were purchased from Sigma-Aldrich (St. Louis, MO, USA). Deionized water from a Simplicity UV water purification system (Millipore, Billerica, MA, USA) was used to make all solutions. The mobile phase which contained 100 mM NaCl and 5 mM Tris-buffer pH 7.4 was prepared by dissolving the appropriate amounts in deionized water and filling to the 1.0 L mark. Mobile phases which contained Cys concentrations between 0.1 and 10 mM were prepared by dissolving the appropriate amounts of Cys in 100 mM NaCl/5 mM Tris-buffer and adjusting the pH to 7.4 with 4.0 M NaOH/HCl using a VWR Symphony SB20 pH meter (Thermo Electron Corporation, Beverly, MA, USA). All mobile phases were filtered through a 0.45 µm pore size filter (Mandel Scientific, Guelph, ON, Canada) before use.

### 2.2. Instrumentation

The HPLC system was comprised of an Azura P2.1S HPLC pump which was equipped with a ceramic pump head, a Rheodyne 9725 injector equipped with a 50 µL sample loop, and a Hamilton PRP-X100 anion-exchange column, Hamilton Company Inc. (Reno, NV, USA; 250 × 4.1 mm I.D., 10 µm particle size). The flow rate was 1.00 mL min^−1^ and 50 µg of Cd was injected. All separations were conducted at room temperature (22 °C) and Cd was detected in the column effluent using a Buck Model 200A flame atomic absorption spectrometer (FAAS, Buck Scientific, East Norwalk, CT, USA) at 228.8 nm. The chromatographic raw data were imported into Sigma Plot 14.5 (Chicago, IL, USA) and smoothed using the bisquare algorithm. Peak areas and retention times were determined using Origin software (Version 2022) (Northampton, MA, USA). All experiments were performed in triplicate to calculate standard deviations unless otherwise stated.

### 2.3. X-ray Spectroscopy Data Collection

Samples and standards were measured at the Stanford Synchrotron Radiation Lightsource (SSRL), California at the beamline 7–3, with the SPEAR electron storage ring containing 500 mA at 3.0 GeV, using a Si (220) double crystal monochromator. Samples were maintained at 10 K using a helium flow cryostat (Oxford instruments, Abingdon, UK) with sample cuvettes inclined at 45° to the incident X-ray beam. For each sample, 7 to 11 scans each of about 35 min duration were accumulated. The incident and transmitted X-ray intensities were monitored using nitrogen-filled gas ionization chambers operating at 1.2 atmospheres with a sweeping voltage of 1600 V, while the Cd K-edge X-ray absorption was measured as the X-ray K_α_ fluorescence excitation spectrum using an array of 30 germanium detectors [24] with Soller slits and six absorption-length silver metal filters to preferentially eliminate scattered X-rays so as to maintain the detector count rates in the pseudo-linear regime. The energy was calibrated with reference to the lowest energy K-edge inflection of cadmium metal foil which was assumed to be 26714.0 eV. Data were collected using the Web version of the XAS Collect data acquisition software (SSRL, Stanford, CA, USA) [25].

### 2.4. X-ray Spectroscopy Data Analysis

Data reduction and analysis were completed using the EXAFSPAK suite [26] of computer programs. The raw data were carefully examined and then calibrated to the strongest inflection point of cadmium foil for Cd K-edge EXAFS. The data were then averaged, and the background function was subtracted using the first-order polynomial over the pre-edge region, followed by the spline calculation which fit through the oscillatory part of the data and extracted the lowest frequency oscillations above the edge and was normalized using the spline method. The extended X-ray absorption fine structure (EXAFS) phase and amplitude functions were calculated using EXAFSPAK program OPT, using feff8.5 [26]. The structural parameters were refined by fitting the k^3^-weighted model function to the experimental EXAFS oscillation, keeping the amplitude reduction factor fixed to 1, and delta E_0_ was fixed to the value of cadmium standard compounds. Other parameters such as the bond length distance (R), and Debye–Waller (σ) parameters were floated accordingly. X-ray absorption near the edge structure spectra of the Cd samples of interest was compared to the structure of previously reported Cd complexes with 2,3-dimercapto-1-propanesulfonic acid (DMPS) and *meso*-2,3-dimercaptosuccinic acid (DMSA) [27].

## 3. Results and Discussion

While the chronic exposure of human populations to toxic metal (loid)s is associated with numerous adverse health effects [28,29], the biomolecular mechanisms which ultimately link these are incompletely understood [9]. One important biological compartment in which relevant toxicological chemistry-related processes unfold that ultimately determine which and how much of a toxic metal and/or its detoxification products impinge on target organs is the systemic blood circulation [9,30]. Although gaining insight into the interaction of toxic metal species with biomolecules in blood plasma is hampered by its complexity, hyphenated chromatographic approaches in combination with X-ray absorption spectroscopy can provide insight into the formation of toxic metal–plasma protein complexes as well as valuable chemical and electronic information of such metal complexes [31,32,33]. Metallomics approaches have also been useful to study the effect of SMW thiols on the stability of toxic metal–plasma protein complexes at near physiological conditions [13,31] and can also provide insight into the coordination behavior of toxic metal ions towards mixtures of biological SMW thiols [34]. 

**Figure 1:**
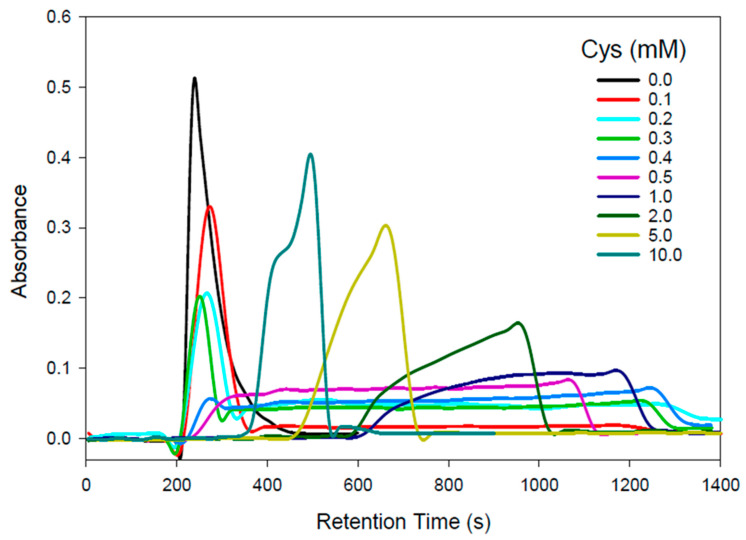
Representative Cd-specific chromatograms obtained by HPLC-FAAS for the analysis of Cd^2+^ (50 µg) on a PRP-X100 anion-exchange column (250 mm × 4.1 mm ID; 10 µm particle size) with a 100 mM NaCl mobile phase that contained 5 mM Tris-buffer (pH 7.4). Flow rate: 1.0 mL/min; injection volume: 50 µL (50 µg Cd^2 +^); detector: flame atomic absorption spectrometer at 228.8 nm.

To investigate the complex formation of Cd^2+^ with physiologically relevant Cys concentrations in 100 mM NaCl/5 mM Tris-buffer (pH 7.4), an AEX-HPLC-FAAS system was employed. The effect of increasing Cys mobile phase concentrations on the retention behavior of Cd is depicted in Figure 1 and the retention times of the corresponding Cd peaks/recoveries (87–107%) in Table 1.

With the 100 mM NaCl/5 mM Tris-buffer (pH 7.4) mobile phase, a single Gaussian-shaped Cd peak eluted (Figure 1, black line), which likely corresponds to [CdCl_3_]^−^ and [CdCl_4_]^2−^ species, which were marginally retained on the anion-exchange column owing to the comparatively high mobile phase Cl^−^ concentration. In contrast, the utilization of a 0.1 mM Cys mobile phase resulted in a single Cd peak that had a 33 s increased retention time compared to the Cys-free mobile phase and was followed by an exceedingly broad ~800 s wide Cd peak (Figure 1, red line). The slightly reduced overall Cd recovery of 87% is attributed to the unusual width of the second Cd peak. Importantly, these chromatographic results imply that in the presence of 0.1 mM Cys in 100 mM Cl^−^, Cd forms two distinct solution species. The marginally retained Cd species possibly corresponds to the positively charged species [CdCys]^+^ and/or the neutral [Cd(Cys)_2_] species, while the strongly retained Cd peak likely corresponds to a negatively charged Cd species, such as [Cd(Cys)_3_]^−^ and/or [Cd(Cys)_4_]^2−^. 

When the Cys mobile phase concentration was increased to 0.2 mM (Figure 1, light blue line), the intensity of the first Cd peak was reduced by ~1/3 and the intensity of the rather broad Cd peak increased about three-fold compared to the 0.1 mM Cys mobile phase. While the results obtained with a 0.3 mM Cys mobile phase (Figure 1, dark green line) revealed rather similar results to those for the 0.2 mM Cys mobile phase, the results for the 0.4 mM Cys mobile phase displayed essentially a single 800 s wide Cd peak (Figure 1, dark blue line), which may correspond to a mixture of [Cd(Cys)_3_]^−^ and [Cd(Cys)_4_]^2−^ complexes. With a 0.5 mM Cys mobile phase, the results resembled those obtained for the 0.4 mM Cys mobile phase, but the Cd peak width was only 600 s, which concomitantly increased its intensity (Figure 1, purple line). These Cd results can be rationalized by the higher Cys concentration of the mobile phase, which more effectively competes with the on-column formed [Cd(Cys)_3_]^−^ and [Cd(Cys)_4_]^2−^ for the positive charges on the stationary phase. A further increase in the mobile phase Cys concentration to 1.0, 2.0, 5.0, and 10.0 mM progressively reduced the retention times of the observed major Cd peak to 1162 s, 949 s, 659 s, and a Cd double peak with retention times of 428 and 492 s (Cd recoveries 87–107%). The observation that with the 10 mM Cys mobile phase two Cd peaks were observed is rationalized by the elution of [Cd(Cys)_3_]^−^ followed by [Cd(Cys)_4_]^2^, which, owing to the additional negative charge on the latter species, is more strongly retained. 

**Figure 2:**
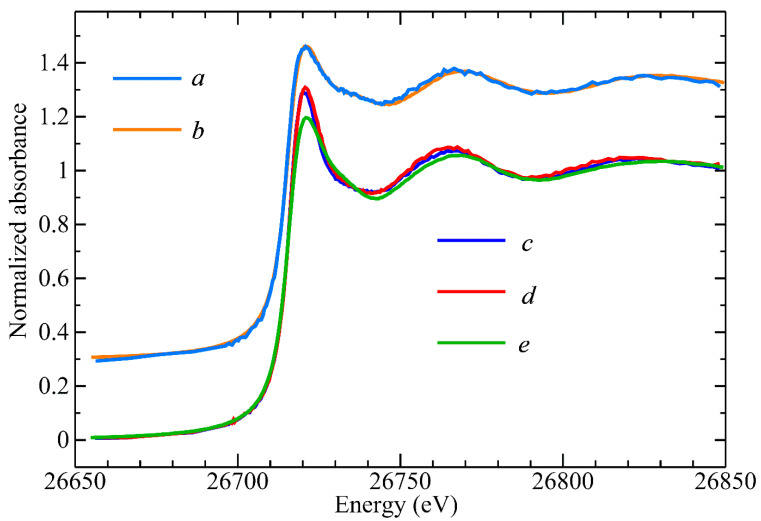
Comparison of Cd K near-edge spectra. (top) Cd^2+^ complex obtained with 200 µM Cys (*a*) compared with 1:1 ratio of Cd^2+^ with DMSA standard (*b*), (bottom) Cd complex obtained with 100 µM Cys (*c*), and Cd complex obtained with Tris-buffer (*d*) compared with 1:0.5 ratio of Cd^2+^ with DMPS standard (*e*).

The rather intriguing Cd-retention behavior on the AEX-HPLC column with increasing Cys mobile phase concentrations can be rationalized by three contributing factors, namely, the Cys-mediated formation of multiple CdCl_x_Cys_y_ species, their different stability in solution, and the competition of the increasing Cys mobile phase concentration with the on-column formed CdCl_x_Cys_y_ species for the positively charged sites of the AEX-HPLC column. The most important chromatographic results from a toxicological point of view are those obtained with Cys concentrations of 0.1 and 0.2 mM Cys mobile phases as these are physiologically relevant [17]. Accordingly, the Cd complexes that eluted with these mobile phases were collected for structural characterization and analysis by X-ray absorption spectroscopy (XAS). The Cd concentration in these samples was close to 30 μM, which is challengingly dilute for XAS, and other workers have suggested that a practical lower limit for sample concentrations is more than an order of magnitude higher than this [35]. The concentration limitations for recording near-edge spectra are less stringent since Cd spectra at a sample concentration of 7 μM have been previously reported [36]. The near-edge spectra of the eluted Cd complexes are shown in Figure 2 together with selected standard compounds.

**Figure 3:**
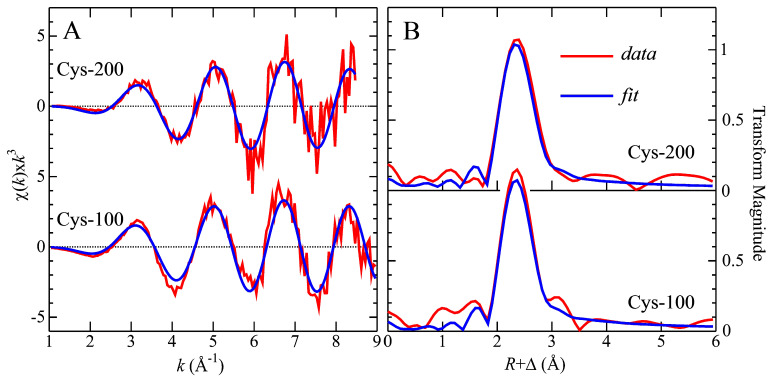
EXAFS spectra (**A**) and corresponding Fourier transforms (**B**) for Cd K-edge for the Cd complex obtained with 100 µM Cys and the Cd complex obtained with 200 µM Cys. The Fourier transforms are phase-corrected for the first shell interactions and bond length distances are shown in Table 2.

The Cd near-edge spectra of the Cd–Cys complex collected from the 0.1 mM Cys mobile phase and the Cys-free mobile phase were rather similar and both were remarkably similar to the corresponding spectra for a Cd^2+^ complex with 2,3-dimercaptopropane-1-sulfonic acid (DMPS) at a molar ratio of 1:0.5, where Cd was bound to one S-atom and three O-atoms [27]. On the other hand, the near-edge spectra for the other Cd–Cys complex, collected from the 0.2 mM Cys mobile phase, and the 1:1 ratio of the Cd^2+^-DMSA model compound were similar. Such results indicate that similar species were formed, suggesting that the metal site has tetrahedral-type symmetry. The Cd K-edge EXAFS spectra, EXAFS curve-fitting analysis, and the corresponding Fourier transforms for these two samples are shown in Figure 3 with the numerical results of the curve fitting given in Table 2.

The curve fitting of the Cd–Cys complex obtained from the 0.1 mM Cys mobile phase showed a definitive 1.5 Cd-S/Cl and 2.5 Cd-O contacts at 2.55 and 2.33 Å, respectively, indicating a 4-coordinate Cd complex, with a mixture of species. EXAFS is unable to directly distinguish backscatterers with close atomic numbers such as Cl and S; however, when backscatterers have different atomic numbers such as S and O, the structural information obtained is very accurate. Moreover, the Fourier transform of the Cd–Cys complex that eluted with the 0.2 mM Cys mobile phase is dominated by a single shell comprising 2 Cd-S and 2 Cd-O ligands at a mean distance of 2.53 and 2.28 Å, respectively, which agrees with the near-edge spectra and bond length distances previously reported for DMSA:Cd at a 1:1 molar ratio as shown in Table 2. In this model compound, the Cd–S interatomic distance is 2.55 Å, and for Cd–O is 2.33 Å, forming a tetrahedral coordination with two cysteinyl and two oxygen ligands to the metal, with the latter presumably coming from water molecules. By comparing these values to the data obtained for the Cd–Cys complex, our experimental values fit at the same atom–atom distance. Overall, in support of these results, the bond-length distances of the structures of Cd coordinated to S and O in different Cd complexes found in the Cambridge Structural Database (CSD) [19,27], are reported in Table 3 and are in good agreement with our experimental data. The Cd concentration of our samples at ~30 µM is close to physiologically relevant levels, but is in the ultra-dilute regime for XAS, which makes these experiments especially challenging. Moreover, the low concentrations mean that other structurally sensitive spectroscopic methods, such as ^113^ Cd nuclear magnetic resonance, cannot easily be applied. Because of this data collection, a significantly shorter-than-normal *k*-range (ca. *k*_max_~9 Å ^−1^) was possible, which under other circumstances would be unacceptable. It is important to note that Cd-Cl ligation would a priori be difficult to distinguish from Cd-S ligation, except that chloride, again with reference to the CSD [18,32], tends to have systematically shorter bond lengths, by about 0.07 Å for similar coordination types (i.e., comparing only four-coordinate species, and not four-coordinate with six-coordinate, as in Table 3). Thus, while it is not possible for us to rigorously eliminate the possibility of chloride ligation, our bond lengths are more consistent with thiolate ligation.

In vivo studies have revealed that Cd^2+^ is rapidly translocated from the bloodstream to its toxicological target organs [14], but the underlying bioinorganic processes are incompletely understood [37,38]. In rats, for example, the temporal analysis of blood plasma for an RSA–Cd complex after their intravenous injection with Cd^2+^ (0.4 mg Cd/kg body wt) revealed its translocation to organs to be complete within 30 min [39]. To gain insight into the dynamic biomolecular processes which orchestrate the translocation of Cd^2+^ to target organs, one needs to consider all ligands that are present in plasma and have an affinity for this metal ion, which include plasma proteins (e.g., HSA); but also ions, such as Cl^−^ which is present at 105 mM; as well as SMW thiols, such as Cys and others [14]. Using the 0.1 mM Cys mobile phase, our EXAFS results revealed the formation of a 1.5 Cd-S/Cl with 2.5 Cd-O species, while the 0.2 mM Cys mobile phase revealed the formation of a 2 Cd − S and 2 Cd-O species. Both Cd species could be involved in the organ uptake of Cd from the bloodstream. While plasma proteins, such as HSA, play a role in the Cd translocation from the bloodstream to the surface of target organ cells [40], it is possible that the formation of mixed Cd complexes with Cl^−^ and Cys—which are formed at near physiological conditions—are potentially also involved. To this end, previous studies have demonstrated that the SMW thiol homocysteine appears to play a role in the translocation of Cd^2+^ to target organs [41], and the same thiol is implicated in the uptake of CH_3_Hg^+^ across the blood-brain barrier (BBB) [42]. Further studies are needed to identify which of the structurally characterized Cd complexes are the actual substrates for uptake mechanisms located at target organs to pave the way for establishing the entire sequence of biomolecular events, which deliver this toxic metal species to the intracellular sites where organ damage is known to unfold [43,44].

## 4. Conclusions

A liquid chromatography-based approach complemented by X-ray absorption spectroscopy was employed to probe the on-column formation of Cd^2+^ complexes with increasing Cys concentrations in a mobile phase that resembled protein-free blood plasma. In contrast to the elution of a single Cd peak near the void volume with a Cys-free mobile phase which may correspond to [CdCl_3_]^−^ and [CdCl_4_]^2−^, the utilization of 0.1–0.4 mM Cys mobile phases revealed a progressive decrease in this Cd peak followed by a second, exceedingly broad Cd peak, which may correspond to [Cd(Cys)_3_]^−^ and [Cd(Cys)_4_]^2−^, that were retained. With the 0.5 mM Cys-containing mobile phase, only the broad Cd peak, possibly a mixture of [Cd(Cys)_3_]^−^ and [Cd(Cys)_4_]^2−^ complexes, was observed whose retention time progressively decreased at Cys concentrations of 1.0, 2.0, 5.0, and 10.0 mM. Our Cd K-edge EXAFS data, together with the Cd K near-edge spectra of the Cd complex species that eluted with the 0.1 and the 0.2 mM Cys-containing mobile phases, revealed a mixture of Cd species with a predominantly tetrahedral coordination of Cd by sulfur and oxygen molecules and an increasing sulfur coordination on the Cd center with an increased Cys concentration. Since any gastrointestinally absorbed toxic metal species is likely to exert toxic effects at target organs if it is absorbed therein, the identified [Cd(Cys)_1/2_]^0/+1^ species are likely to be of direct toxicological relevance. The obtained results are therefore relevant in the context of delineating the entire sequence of biomolecular processes which causally link human exposure to Cd with adverse health effects and possibly diseases, such as type 2 diabetes [45]. Further research is necessary to establish if Cd–L-cysteine complexes are substrates for the uptake mechanisms that are located at target organs in order to causally link the human exposure to Cd with human diseases of unknown etiology [46,47,48,49], and to deepen our understanding of the bioinorganic chemistry that unfolds at the blood plasma–red blood cell–organ nexus [30,50].

## Figures and Tables

**Table 1:** Results obtained after the analysis of Cd^2+^ (50 µg) on an HPLC-FAAS system using mobile phases that contained 100 mM of NaCl, 5.0 mM Tris-buffer (pH 7.4), and increasing concentrations of cysteine (Cys) (0 to 10 mM) using a Hamilton PRP-X100 (AEX) HPLC column at flow rate 1.0 mL/min and a Buck Scientific FAAS as the Cd-specific detector at 228.8 nm.

[Cys] in Mobile Phase (mM)	Retention Time (s) ^+^	Cd Recovery (%)
0	239 ± 0	100
0.1	272 ± 1 * 418 ± 24 1136 ± 47	87 ± 2
0.2	263 ± 2 * 373 ± 8 1210 ± 72	89 ± 8
0.3	248 ± 2 * 327 ± 2 1216 ± 8	95 ± 3
0.4	269 ± 4 * 460 ± 27 1247 ± 3	94 ± 18
0.5	1057 ± 7 * 565 ± 7 437 ± 4	107 ± 6
1	1035 ± 12 1162 ± 8 *	101 ± 3
2	949 ± 19	97 ± 3
5	659 ± 2	100 ± 3
10	428 ± 9 492 ± 4 *	96 ± 3

^+^ *n* = 3. * Main, largest peak.

**Table 2:** Summary EXAFS curve-fitting parameters for Cd compounds.

Samples	Bond	*N*	R (Å)	σ^2^ (Å^2^)	ΔE_0_ (eV)
Cd-0.1 mM Cys	Cd-S/Cl Cd-O	1.5 2.5	2.556 2.334(6)	0.0024(8) 0.0045(12)	−11.17
Cd-0.2 mM Cys	Cd-S	2	2.547(5)	0.0034(16)	−11.17
	Cd-O	2	2.333	0.0077(48)	
Standard compounds reported previously [27]					
DMPS:Cd (molar ratio 1:0.5)	Cd-S	0.9	2.556(15)		
	Cd-O	3.1	2.334(13)		
DMSA:Cd (molar ratio 1:1)	Cd-S	1.8	2.553(7)		
	Cd-O	2.2	2.339(17)		

**Table 3:** Average of bond lengths and angles for relevant structures surveyed from the Cambridge Structural Database.

Fragment	*n* [a]	Bond	Distance [b]	Fragment	*n* [a]	Bond	Distance [b]
CdO_6_	597	Cd-O	2.287(68)	CdO_2_S_4_	14	Cd-O Cd-S	2.458(123) 2.651(45)
CdS_6_	36	Cd-S	2.708(60)	CdO_4_S_2_	44	Cd-O Cd-S	2.330(77) 2.631(58)
CdO_3_S_3_	19	Cd-O Cd-S	2.517(101) 2.568(35)	CdO_5_S_1_	2	Cd-O Cd-S	2.304(43) 2.593(26)
CdO_5_	28	Cd-O	2.272(84)	CdS_5_	104	Cd-S	2.635(87)
CdO_2_S_3_	23	Cd-O Cd-S	2.407(103) 2.555(50)	CdS_3_	10	Cd-S	2.476(43)
CdOS_4_	8	Cd-O Cd-S	2.402(31) 2.615(88)	CdO_3_S_2_	10	Cd-O Cd-S	2.319(77) 2.535(38)
CdO_4_	29	Cd-O	2.180(68)	CdS_4_	554	Cd-S	2.538(32)
CdOS_3_	20	Cd-O Cd-S	2.285(55) 2.507(24)	CdO_2_S_2_	11	Cd-O Cd-S	2.183(46) 2.572(85)
ClCdX3 (any)	888	Cd-Cl	2.459(45)				

ls [a] *n* is the number of occurrences of the fragment in the database, where one database entry may have more than one inequivalent fragment in the structure. [b] Value shows the bond length distance mean ± standard deviation (Å) for the given *n* observations.

## Data Availability

All data are contained within the article.
